# Structural characterisation and antioxidant and prebiotic activities of *Ophiopogon japonicus* polysaccharides obtained by enzymatic pretreatment followed by simultaneous microwave–ultrasound-assisted extraction

**DOI:** 10.3389/fmicb.2026.1927939

**Published:** 2026-09-09

**Authors:** Henan Sun, Hao Yu, Huiqiang Yu, Sarengaowa, Ke Feng, Wenzhong Hu

**Affiliations:** 1 School of Life Sciences, Zhuhai College of Science and Technology, Zhuhai, China; 2 College of Life Sciences, Jilin University, Changchun, China; 3 Medical Sciences Division, Macau University of Science and Technology, Taipa, Macao SAR, China

**Keywords:** antioxidant activity, enzymatic pretreatment, microwave-ultrasound assisted extraction, *Ophiopogon japonicus* polysaccharide, prebiotic activity, structural characterisation

## Abstract

In this study, response surface methodology was used to optimize a sequential two-stage process comprising enzymatic pretreatment followed by simultaneous microwave–ultrasound-assisted extraction of *Ophiopogon japonicus* polysaccharides. At the unrounded model optimum (208.492 W microwave power, 300.945 W ultrasonic power, 25.207 min enzymatic hydrolysis, and 30.100 min microwave–ultrasound extraction), the predicted crude polysaccharide extract yield was 43.999% (44.0% when rounded). Two fractions, OJP-1 and OJP-2, were obtained by DEAE-52 and Sephadex G-100 chromatography. Structural analyses showed differences in their monosaccharide composition, spectral properties, surface morphology, thermal stability, and HPGPC molecular-mass profiles. *In vitro*, OJP-2 showed stronger radical-scavenging and probiotic growth-promoting responses than OJP-1, whereas both fractions resisted hydrolysis in the simulated digestion systems. OJP-2’s stronger responses were accompanied by distinct compositional, morphological, thermal, and HPGPC molecular-mass-profile characteristics.

## Graphical abstract

**Figure:**
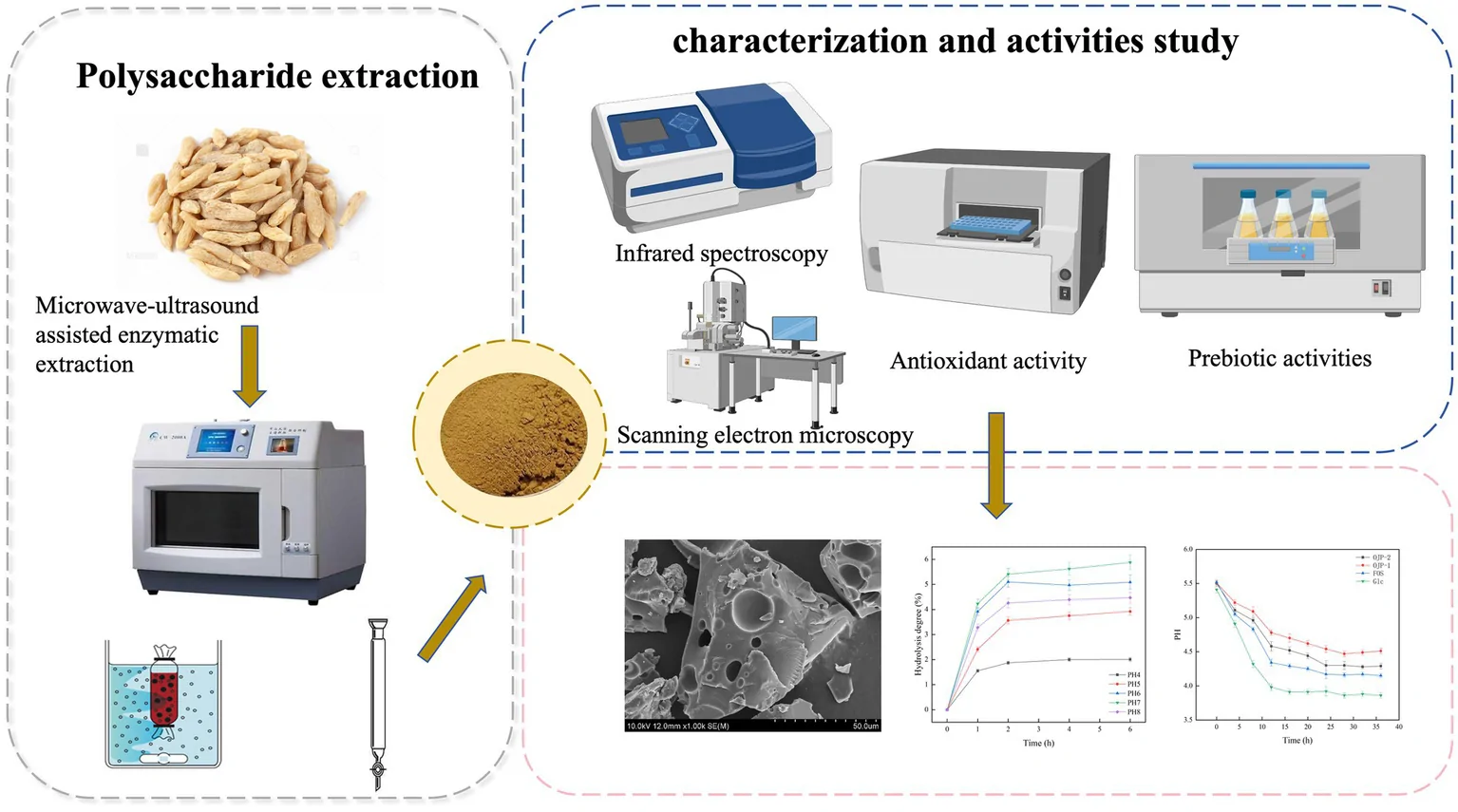

## 1 Introduction

*Ophiopogon japonicus* (L.f.) Ker Gawl. is a species in the family Asparagaceae. Its dried tuberous root is commonly used in traditional Chinese medicine (Lei et al., 2021). *O. japonicus* is rich in polysaccharides, saponins, flavonoids, and various amino acids and trace elements, with polysaccharides being the main active ingredients (Zhang Y. et al., 2024). In traditional Chinese medicine, the root has been used to nourish yin, moisten the lungs, promote fluid production, and relieve dry cough and thirst associated with lung or stomach yin deficiency (Song, 2024). Modern pharmacological studies have reported antioxidant (Wang et al., 2012), intestinal flora-regulatory (Li et al., 2025), immune-regulatory (Lin et al., 2025), and hypoglycaemic properties (Chen et al., 2011). Owing to its medicinal and edible applications, *O. japonicus* is used as a source of functional ingredients.

Plant polysaccharides are highly polar macromolecules that are commonly extracted with water because of their water solubility and ethanol insolubility. Conventional hot-water extraction can require high temperatures and long treatment times (Huang and Huang, 2020). Ultrasound-assisted extraction has therefore been applied to plant polysaccharides (Ye et al., 2025). Ultrasound-assisted enzymatic extraction combines enzymatic hydrolysis with acoustic cavitation and has been reported to improve extraction efficiency (Zhou et al., 2025); ultrasound can alter polysaccharide structures (Peng et al., 2025). Source-specific studies have reported changes in yield, composition, or physicochemical properties after ultrasound-assisted enzymatic extraction (Lin et al., 2023; Olawuyi et al., 2020). Polysaccharide structural features have been associated with gut-microbiota responses (Li et al., 2022; Song et al., 2023; Sun et al., 2024), and inulin is a growth-promoting prebiotic substrate (Kaewarsar et al., 2023). Process effects depend on the source material and extraction conditions (Gao et al., 2025). In the present process, enzymatic hydrolysis was completed before microwave and ultrasound were applied simultaneously; the process is termed enzymatic pretreatment followed by simultaneous microwave–ultrasound-assisted extraction.

In this study, the sequential two-stage extraction process and response surface methodology (RSM) were used to optimize crude polysaccharide extract yield. The resulting material was purified into OJP-1 and OJP-2 and characterized using compositional, spectroscopic, chromatographic, microscopic, and thermal analyses. The fractions were then evaluated for DPPH, ABTS, and hydroxyl-radical scavenging, reducing power, stability in artificial saliva and gastric juice, and growth-promoting effects on *Lactobacillus plantarum*, *Lactobacillus acidophilus*, and a mixed culture.

## 2 Materials and methods

### 2.1 Materials and reagents

The roots of *Ophiopogon japonicus* were purchased from Mianyang, Sichuan. Analytical-grade anhydrous ethanol, chloroform, n-butanol, hydrogen peroxide, and phenol were purchased from Sinopharm Chemical Reagent Co., Ltd. (Beijing, China). Analytical grade glucose, glucuronic acid, and Coomassie Brilliant Blue dye were purchased from Aladdin Biochemical Technology Co., Ltd. (Shanghai, China). Sodium tetraborate, carbazole, FOS, papain, DPPH, salicylic acid, potassium bromide (KBr), artificial gastric juice, and artificial saliva were purchased from Shanghai Yuanye Biotechnology Co., Ltd. (Shanghai, China). Concentrated sulfuric acid, sodium sulfate, potassium persulfate, ferrous sulfate, potassium ferricyanide, trichloroacetic acid, trifluoroacetic acid, and ferric chloride were purchased from Shanghai Aladdin Biochemical Technology Co., Ltd. (Shanghai). The 3,5-dinitrosalicylic acid (DNS) reagent and lactate assay kit were purchased from Beijing Solebao Technology Co., Ltd. (Beijing, China).

### 2.2 Extraction and purification of crude polysaccharides

*O. japonicus* from Mianyang, Sichuan was selected as the raw material, dried in a drying oven to a constant weight, crushed using a high-speed pulveriser and screened through a 20-mesh sieve to obtain a powder with a uniform particle size.

For each extraction, 10.0 g of dry *O. japonicus* powder was mixed with 100 mL of distilled water (solid-to-liquid ratio, 1:10, g:mL). Papain was added at 2% (w/w, relative to the dry raw-material mass), and no pH adjustment was performed. Enzymatic hydrolysis was first performed for the time assigned to each experiment. Microwave and ultrasound were then applied simultaneously at 60 °C, at the assigned powers and for the assigned duration of the microwave–ultrasound extraction stage. Immediately after extraction, the mixture was cooled in a cold-water bath to terminate the treatment and centrifuged at 8,000 rpm for 6 min. The supernatant was concentrated under reduced pressure using a rotary evaporator. Anhydrous ethanol was then added to a final ethanol concentration of 80% (v/v), and the mixture was left overnight. The precipitate was collected by filtration and dried at 60 °C to constant mass. This oven-dried ethanol precipitate was defined as the crude polysaccharide extract for gravimetric yield determination.

The crude polysaccharide extract yield was calculated as follows:


$$\text{Crude polysaccharide extract yield}\;(\%)=\frac{m_{\text{full}}-m_{\text{empty}}}{m_{0}}\;x\;100$$

Where m_full is the mass of the weighing container plus the oven-dried 80% ethanol precipitate, m_empty is the mass of the empty container, and m_0 is the dry mass of the starting *O. japonicus* powder. Yield was determined gravimetrically from the oven-dried 80% ethanol precipitate before purification. This pre-purification precipitate was not purity-corrected for carbohydrate content and is reported throughout as crude polysaccharide extract yield.

For downstream purification, the crude extract was redissolved and deproteinised with Sevage reagent (chloroform:n-butanol, 4:1, v/v) at a crude-extract solution: Sevage ratio of 1:1 (v/v). The treatment was repeated until no protein layer was visible at the phase interface. When phase separation was incomplete, the mixture was centrifuged at 6,000 rpm for 10 min. The aqueous phase was dialysed in a 2,000-Da molecular-weight-cutoff bag against running water for 48 h, frozen at −20 °C, and lyophilised. These downstream purification steps were not included in the extraction-yield calculation.

For column purification, 20 mL of the lyophilised sample redissolved at 25 mg/mL was loaded onto a DEAE-52 cellulose column (2.0 × 20 cm). The column was eluted sequentially with distilled water, 0.1 M NaCl, and 0.3 M NaCl. Fractions were monitored using the phenol–sulfuric acid method, pooled according to their elution profiles, concentrated, desalted, and lyophilised. The first two fractions were further purified on a Sephadex G-100 column (1.5 × 75 cm), yielding OJP-1 and OJP-2 after lyophilisation.

### 2.3 Single-factor experimental design

A single-factor design was used to determine the preliminary range of extraction factors: A (microwave power, 100–500 W), B (ultrasonic power, 100–500 W), C (enzymatic hydrolysis time, 15–35 min), and D (simultaneous microwave–ultrasound extraction time, 20–40 min). Crude polysaccharide extract yield was the response variable.

### 2.4 Response surface experimental design

The impact of the four variables was studied using RSM. Table 1 lists the variables and levels of the Box–Behnken design (BBD).

**Table 1:** Variables and levels for Box–Behnken design.

Variable	Symbols	Coded levels		
	Coded	−1	0	1
Microwave power(W)	A	100	200	300
Ultrasonic power(W)	B	200	300	400
Enzymatic hydrolysis time(min)	C	20	25	30
Simultaneous microwave–ultrasound extraction time (min)	D	25	30	35

Variables A, B, C and D represent microwave power, ultrasonic power, enzymatic hydrolysis time, and simultaneous microwave–ultrasound extraction time, respectively. Levels (−1), (0), and (+1) represent low, medium, and high levels of the variables, respectively.

### 2.5 Chemical composition analysis

#### 2.5.1 Determination of total sugar, protein and glucuronic acid content

The total sugar content of OJP-1 and OJP-2 was measured using the phenol-sulfuric acid technique (Song et al., 2024); the protein content was determined using the Coomassie Brilliant Blue method (Chen et al., 2018); and the uronic acid content was determined using the carbazole-sulfuric acid method (Huang and Lin, 2024).

### 2.6 Structural characterisation

#### 2.6.1 Ultraviolet–visible (UV–vis) and Fourier transform infrared spectroscopy (FT-IR) analysis

Full wavelength scanning was performed over a range of 200 to 400 nm using a UV–vise spectrophotometer (Beijing Puxi General Instrument Co., Ltd., Beijing, China).

The KBr tableting method was used to obtain the infrared spectrum of the samples. KBr was mixed with the samples separately and pressed into tablets. The vibrational region of 400–4,000 cm^−1^ was scanned with FT-IR (Nexus IS10 FT-IR, Thermo Fisher Scientific).

#### 2.6.2 HPGPC molecular-mass analysis

HPGPC molecular-mass analysis of OJP-1 and OJP-2 was performed on an Agilent 1,100 Series GPC-HPLC system equipped with an RI detector and a TSK-gel G3000 PWXL column (7 μm, 7.8 × 300 mm; TOSOH) (Zhang N. et al., 2024). Samples were membrane-filtered and analyzed with a 20 μL injection. The column was maintained at 40 °C, with 0.05 M sodium sulfate as the mobile phase at 0.5 mL/min. Dextran standards (180–300,600 Da) were used for calibration, with tR = −2.3944 log(Mw) + 23.289 (R^2^ = 0.9930). Molecular-mass results are reported as laboratory-reported, peak-resolved Mw values.

#### 2.6.3 Analysis of monosaccharide composition

Pre-column derivatisation combined with reversed-phase high-performance liquid chromatography was used to analyze the monosaccharide composition of OJPs (Fang et al., 2020). An appropriate amount of sample was mixed with 1 mL of trifluoroacetic acid (4 M TFA) in a tube. The tube was sealed with a spray gun, and its contents were hydrolysed in an oven at 110 °C for 4 h, followed by neutralization with NaOH. To 200 μL of solution, 200 μL of internal standard was added, and a 100 μL aliquot of the mixture was withdrawn and mixed with 100 μL of 0.3 M NaOH and 0.5 M 1-phenyl-3-methyl-5-pyrazolinone (PMP), followed by heating in a water bath set at 70 °C for 1 h in the dark. The mixture was subsequently cooled to 28 °C and neutralized with 100 μL of 0.3 M HCl. It was then extracted with 500 μL chloroform and centrifuged at 7000 rpm for 5 min. The upper layer was aspirated, and the extraction was repeated four times. Chromatography was performed on a column of Agilent Eclipse XDB-C18 (5 μm, 4.6 × 250 mm), with gradient elution. The composition of the mobile phases was as follows: mobile phase A, 18% acetonitrile triethylamine; mobile phase B, 60% acetonitrile triethylamine. The flow rate was 1 mL/min, column temperature was 30 °C, and detection wavelength was 254 nm.

#### 2.6.4 Analysis of the triple-helix structure

The Congo red test was used to evaluate the presence of a triple-helix structure in the sample (Zhang W. et al., 2020). Equal volumes of polysaccharide solution (1 mg/mL) were mixed with Congo red solution (80 μmol/L), and an appropriate amount of NaOH solution (1 mol/L) was added at a final concentration of 0–0.5 M. UV spectrophotometery was performed over a wavelength range of 400–600 nm to determine the absorption maximum.

#### 2.6.5 X-ray diffractometer (XRD) analysis

The crystal structures of OJP-1 and OJP-2 were analyzed using an XRD. The scanning rate was 5°/min, and the 2θ diffraction angle ranged from 5° to 80° (Dou et al., 2023).

#### 2.6.6 Scanning electron microscopy (SEM)

One milligram of each crushed sample was completely dissolved in deionized water. The solution was dropped onto a silicon wafer and sputter-coated with gold for 15 min. Surface morphology was observed by scanning electron microscopy at 0.5–30 kV and ×500 and ×1,000 magnification.

#### 2.6.7 Atomic force microscopy (AFM)

A 5-μL aliquot of polysaccharide solution (5 mg/mL) was placed on a mica sheet and allowed to dry. Atomic-force-microscopy images were acquired over a 5 μm × 5 μm scan area to characterize surface topography.

### 2.7 Thermogravimetric analysis (TGA)

TGA was performed using a DTG-60H thermogravimetric analyser (Shimadzu Corporation, Japan), with the temperature increasing from 30 °C to 800 °C at a rate of 20 °C/min, to detect the mass loss rate of polysaccharide samples (Mimmo et al., 2005).

### 2.8 Evaluation of antioxidant capacity *in vitro*

The *in vitro* antioxidant activities of OJP-1 and OJP-2 were evaluated by measuring their ABTS, DPPH, and •OH-scavenging activities, and total reducing capacity.

The ABTS radical-scavenging activity was determined using a previously reported method, with slight modifications (Liu et al., 2023). Ten milliliter of ABTS solution (2 mM) was mixed with 100 μL of potassium persulphate solution (70 mM) and stored at 28 °C in the dark for 16 h. One hundred microlitre of the diluted extract was mixed with 1 mL of ABTS solution, and the absorbance of the mixture was read at 734 nm.

The DPPH radical-scavenging activity was evaluated using a previously described method, with slight modifications (Zhang et al., 2016). The diluted extract (1 mL) was mixed with 2 mL of DPPH solution (0.2 mM) using a vortex mixer, and the absorbance of the mixture was read at 517 nm.

For detecting •OH-scavenging ability, we followed a previously reported method (Ding et al., 2023). To 1 mL of different concentrations of OJP solution, 1 mL each of FeSO_4_ (9 mmol/L) and salicylic acid (9 mmol/L) were mixed, and the absorbance of the mixture was read at 510 nm.

The total reducing power was estimated using slightly modified version of the method reported previously (Premarathna et al., 2024). To 1 mL of polysaccharide solution, 0.5 mL of 1% potassium ferricyanide solution, 0.2 mL of 0.2 mol/L phosphate buffer (pH 6.6), 1 mL of 10% trichloroacetic acid, 10 μL of 1% ferric chloride solution, and 150 μL of distilled water were added. The absorbance of the mixture was determined at 700 nm.

### 2.9 Evaluation of *in vitro* polysaccharide digestion characteristics under simulated saliva and gastric juice conditions

*In vitro* digestion experiments were conducted using OJP-1 and OJP-2. Simulated salivary digestion was performed on polysaccharide samples (OJP-1 and OJP-2) using the method described previously, with slight modifications (Yan et al., 2022; Yuan et al., 2020). For the salivary digestion experiment, the pH of the artificial saliva was adjusted to 4, 5, 6, 7, and 8. Equal volumes of 10 mg/mL OJP-1, OJP-2, and positive control fructooligosaccharides (FOS) solutions were mixed with artificial saliva at different pH values and incubated for 6 h to simulate the oral conditions. The mixtures were incubated at 37 °C for another 6 h, and samples were taken at 0, 1, 2, 4, and 6 h. The samples were immediately boiled at 100 °C to inactivate the enzymes, and the reducing sugar content was quantified using the DNS method. The total sugar content was determined using the phenol-sulphuric acid method and the degree of polysaccharide hydrolysis was calculated.

Polysaccharide samples (OJP-1 and OJP-2) were subjected to simulated gastric digestion using the previous described method, with slight modifications (Yan et al., 2022; Yuan et al., 2020; Yu et al., 2024). Gastric juice was mixed with an equal volume of salivary digestive fluid, and the pH of the simulated gastric juice was adjusted to 1, 2, 3, 4, and 5. Samples were taken at 0, 1, 2, 4, and 6 h and immediately boiled at 100 °C to inactivate the enzymes. The reducing and total sugar content was subsequently determined.

### 2.10 Evaluation of the prebiotic activity *in vitro*

In this study, three strains (*L. plantarum*, *L. acidophilus*, and mixed bacteria) were used to evaluate the proliferative effects of polysaccharides. OJP-1, OJP-2, glucose, and FOS were added to MRS sugar-free medium at mass-volume concentrations of 0, 0.5, 1, 1.5, 2, and 2.5% (w/v), respectively. Subsequently, each of the three probiotic strains was inoculated into the culture medium and cultured with shaking at 37 °C for 48 h. The optimal polysaccharide addition level was determined by measuring the OD values of cultures under different polysaccharide concentrations.

All probiotic cultures were performed in a carbon dioxide bacterial incubator. The strains were inoculated into MRS medium supplemented with different 2% (w/v) carbon sources (glucose, FOS, OJP-1, and OJP-2). MRS basal medium was used as a blank control group, and medium containing glucose and FOS was used as a positive control group. All groups were cultured under anaerobic conditions at 37 °C for 48 h, and the absorbance at 600 nm (OD_600_) and pH were measured at different time periods (Fu et al., 2024). The content of reducing sugars was measured using the DNS method, and the lactic acid content was measured according to the instructions provided in the lactic acid determination kit.

### 2.11 Statistical analysis

Data are presented as mean ± standard deviation where replicate measurements were available. Statistical analyses were performed using SPSS and Origin 2024 software; Design-Expert 13 was used for the Box–Behnken design, response-surface model fitting, and optimisation. Differences between groups were assessed using analysis of variance (ANOVA). A *p* value <0.05 was considered statistically significant.

## 3 Results and discussion

### 3.1 Single-factor experimental analysis

Single-factor experiments showed changes in crude polysaccharide extract yield across microwave power, ultrasonic power, enzymatic hydrolysis time, and simultaneous microwave–ultrasound extraction time.

**Figure 1:**
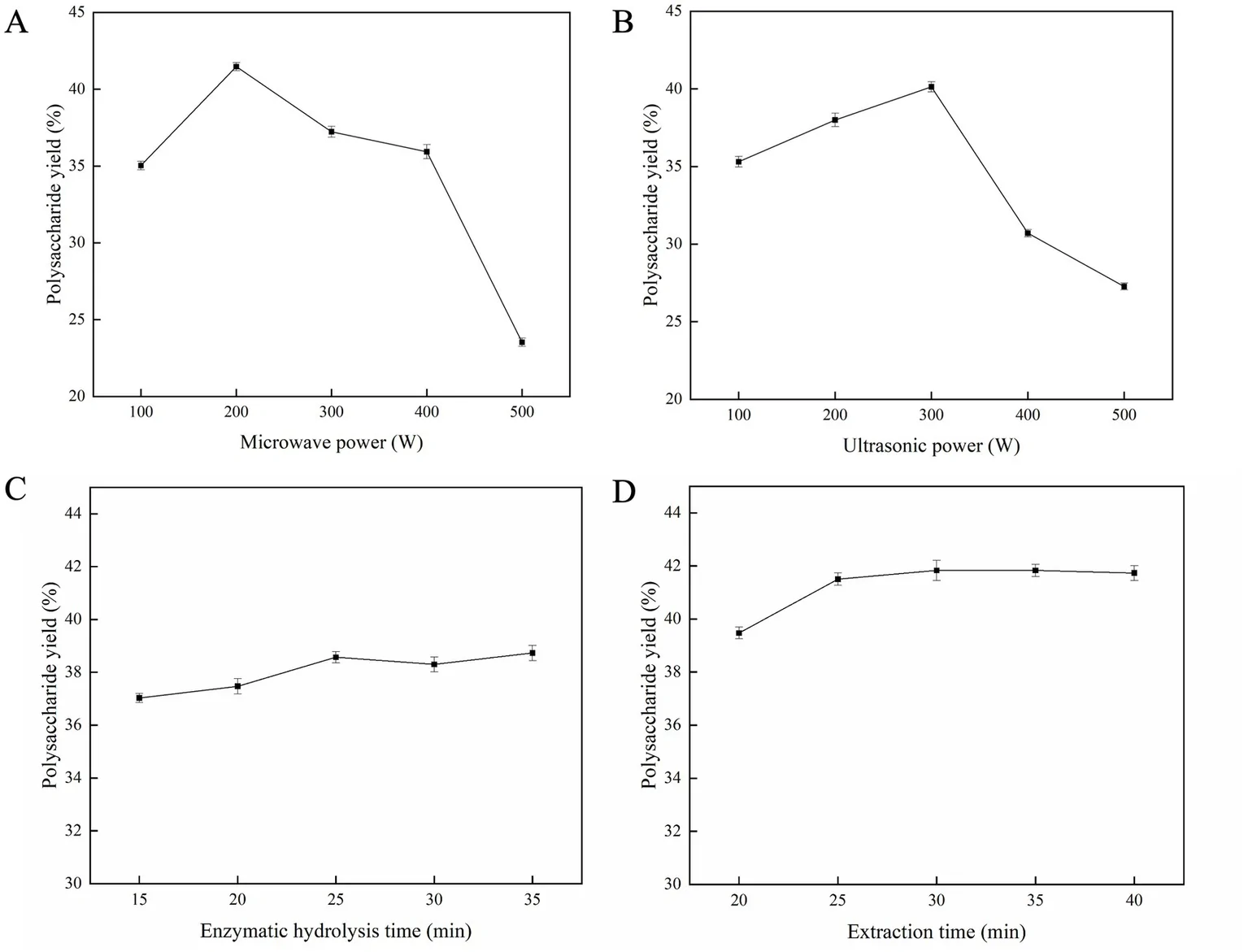
Effects of microwave power **(A)**, ultrasonic power **(B)**, enzymatic hydrolysis time **(C)**, and simultaneous microwave–ultrasound extraction time **(D)** on crude polysaccharide extract yield.

Crude polysaccharide extract yield increased and then decreased as microwave power increased, reaching its highest observed value at 200 W (Figure 1A). The highest observed yield across the ultrasonic-power series occurred at 300 W (Figure 1B), and lower yields were observed at higher powers. Similar power-dependent responses have been reported in polysaccharide extraction (Zhang M. et al., 2020). The decline at high power identified the practical operating range for the extraction process. The highest values in the series for enzymatic hydrolysis time and simultaneous microwave–ultrasound extraction time occurred near 25 and 30 min, respectively (Figures 1C,D), which informed the subsequent response-surface design.

### 3.2 Response surface analysis

Using the 29 Box–Behnken design runs in Table 2, multiple regression analysis gave the quadratic model Y = 43.95 + 0.81A + 0.24B + 0.35C + 0.21D − 1.01AB − 0.38 AC − 0.34 AD − 0.67 BC − 1.12BD − 0.28CD − 4.54A^2^–5.46B^2^–3.70C^2^–4.02D^2^. Here, Y is crude polysaccharide extract yield (%), and A, B, C, and D are coded microwave power, ultrasonic power, enzymatic hydrolysis time, and simultaneous microwave–ultrasound extraction time, respectively.

**Table 2:** Box–Behnken experimental design and crude polysaccharide extract yield.

Run	A	B	C	D	Crude extract yield (%)
1	100	300	20	30	34.08
2	200	300	30	35	36.61
3	200	300	20	25	35.46
4	100	300	25	35	35.03
5	200	300	25	30	44.11
6	100	300	30	30	35.79
7	200	400	30	30	34.62
8	300	300	25	35	35.75
9	300	300	30	30	36.53
10	300	300	20	30	36.35
11	300	200	25	30	35.77
12	200	200	25	35	35.6
13	100	300	25	25	34.27
14	200	400	25	35	33.88
15	200	400	20	30	35.34
16	200	300	25	30	44.11
17	200	200	25	25	32.77
18	200	400	25	25	35.53
19	100	200	25	30	31.82
20	200	300	20	35	36.6
21	200	300	25	30	44.11
22	200	300	25	30	43.88
23	100	400	25	30	34.31
24	300	300	25	25	36.34
25	300	400	25	30	34.23
26	200	300	25	30	43.56
27	200	300	30	25	36.57
28	200	200	30	30	35.49
29	200	200	20	30	33.54

Table 3 summarizes the analysis of variance for the fitted quadratic model. The coefficient of determination (R^2^ = 0.9980) indicated close agreement between fitted and observed responses within the design space.

**Table 3:** Analysis of variance for the BBD quadratic model of crude polysaccharide extract yield.

Source of variance	Sum of squares	Degrees of freedom	Mean square	*F* value	*p* value	Significance
Model	361.67	14	25.83	498.03	< 0.0001	**
A-Microwave power	7.79	1	7.79	150.22	< 0.0001	**
B-Ultrasonic power	0.7105	1	0.7105	13.70	0.0024	**
C-Enzymatic hydrolysis time	1.50	1	1.50	28.88	< 0.0001	**
D-Extraction time	0.5334	1	0.5334	10.28	0.0063	**
AB	4.06	1	4.06	78.27	< 0.0001	**
AC	0.5852	1	0.5852	11.28	0.0047	**
AD	0.4556	1	0.4556	8.78	0.0103	*
BC	1.78	1	1.78	34.36	< 0.0001	**
BD	5.02	1	5.02	96.73	< 0.0001	**
CD	0.3025	1	0.3025	5.83	0.0300	*
A^2^	133.59	1	133.59	2575.44	< 0.0001	**
B^2^	193.34	1	193.34	3727.18	< 0.0001	**
C^2^	88.78	1	88.78	1711.43	< 0.0001	**
D^2^	104.86	1	104.86	2021.57	< 0.0001	**
Residual	0.7262	14	0.0519			
Lack of Fit	0.4925	10	0.0492	0.8429	0.6257	–
Pure Error	0.2337	4	0.0584			
Cor Total	362.40	28				
R^2^	0.9980					
Adjusted R^2^	0.9960					
Predicted R^2^	0.9912					

Factor D denotes simultaneous microwave–ultrasound extraction time; * *p* < 0.05; **p < 0.01; − not significant.

The model was significant (*F* = 498.03, *p* < 0.0001), whereas lack of fit was not significant relative to pure error (*F* = 0.8429, *p* = 0.6257). Among the linear terms, microwave power (A) had the largest *F* value.

**Figure 2:**
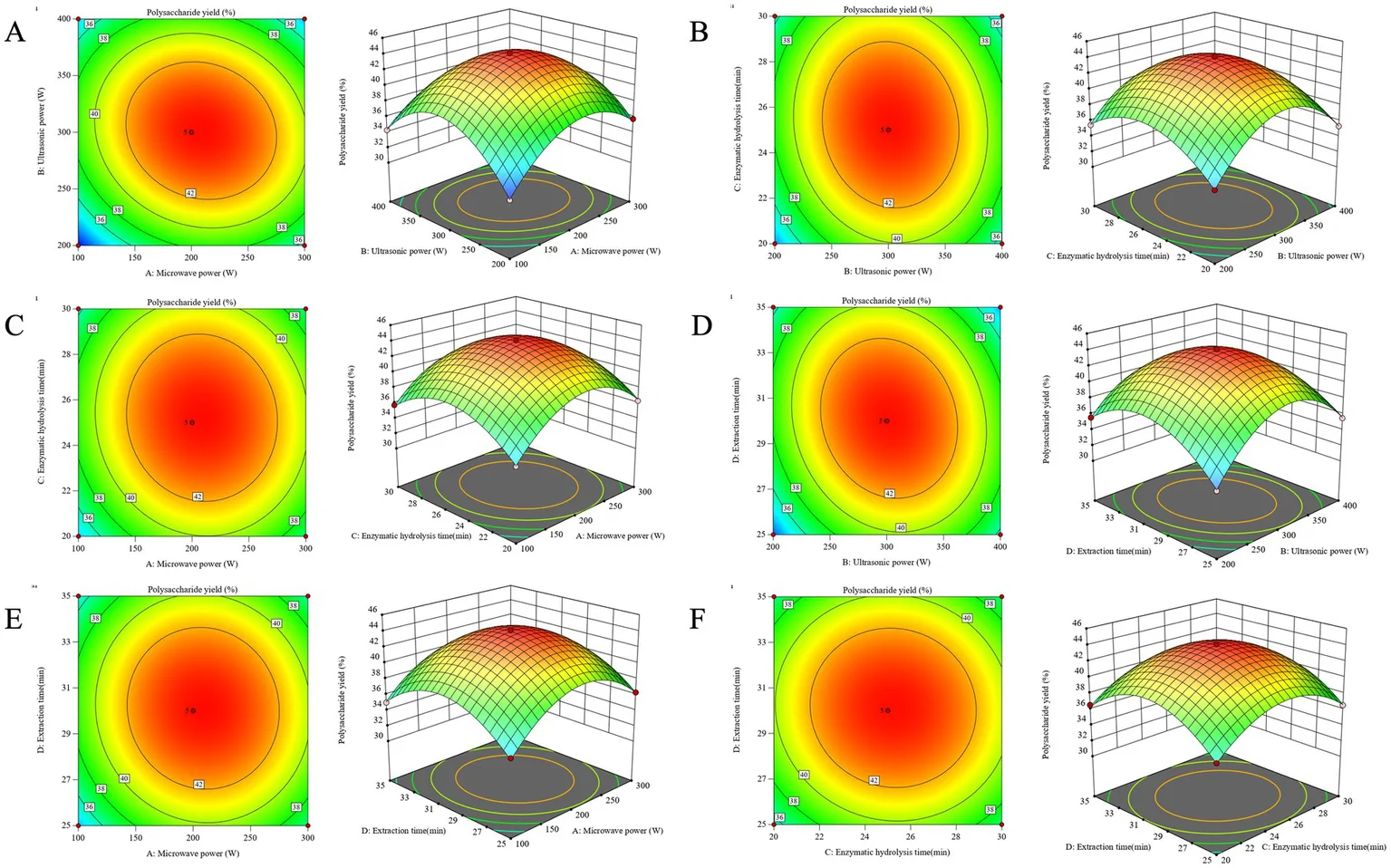
Three-dimensional response surface and two-dimensional contour plots showing the effects of extraction variables on crude polysaccharide yield. **(A)** Microwave power and ultrasonic power; **(B)** ultrasonic power and enzymatic hydrolysis time; **(C)** microwave power and enzymatic hydrolysis time; **(D)** ultrasonic power and extraction time; **(E)** microwave power and extraction time; **(F)** enzymatic hydrolysis time and extraction time.

The fitted surfaces showed an internal maximum within the design space (Figure 2). At the unrounded model optimum (208.492 W microwave power, 300.945 W ultrasonic power, 25.207 min enzymatic hydrolysis, and 30.100 min microwave–ultrasound extraction), the predicted crude polysaccharide extract yield was 43.999%. The fitted model gave a 95% confidence interval of 43.78–44.22% for the mean response and a 95% prediction interval of 43.46–44.53% for one future response. To facilitate practical laboratory operation, the model-derived settings were rounded to whole numbers (208 W microwave power, 300 W ultrasonic power, 25 min enzymatic hydrolysis, and 30 min simultaneous microwave–ultrasound extraction). The fitted prediction at these rounded settings was 43.989% (95% single-response prediction interval, 43.45–44.52%). Five repeated extractions yielded 43.67, 43.39, 43.77, 43.81, and 43.20%, giving 43.57 ± 0.26% (mean ± sample SD; *n* = 5; CV = 0.60%). The observed mean differed from the fitted prediction by 0.42 percentage points (relative error, 0.96%), demonstrating close agreement between the experimental and predicted yields. The low CV further indicates good repeatability at the rounded operating conditions. The significant quadratic model, nonsignificant lack of fit, and high R^2^ values indicate good fit within the investigated design space.

### 3.3 Isolation and purification of OJPs

**Figure 3:**
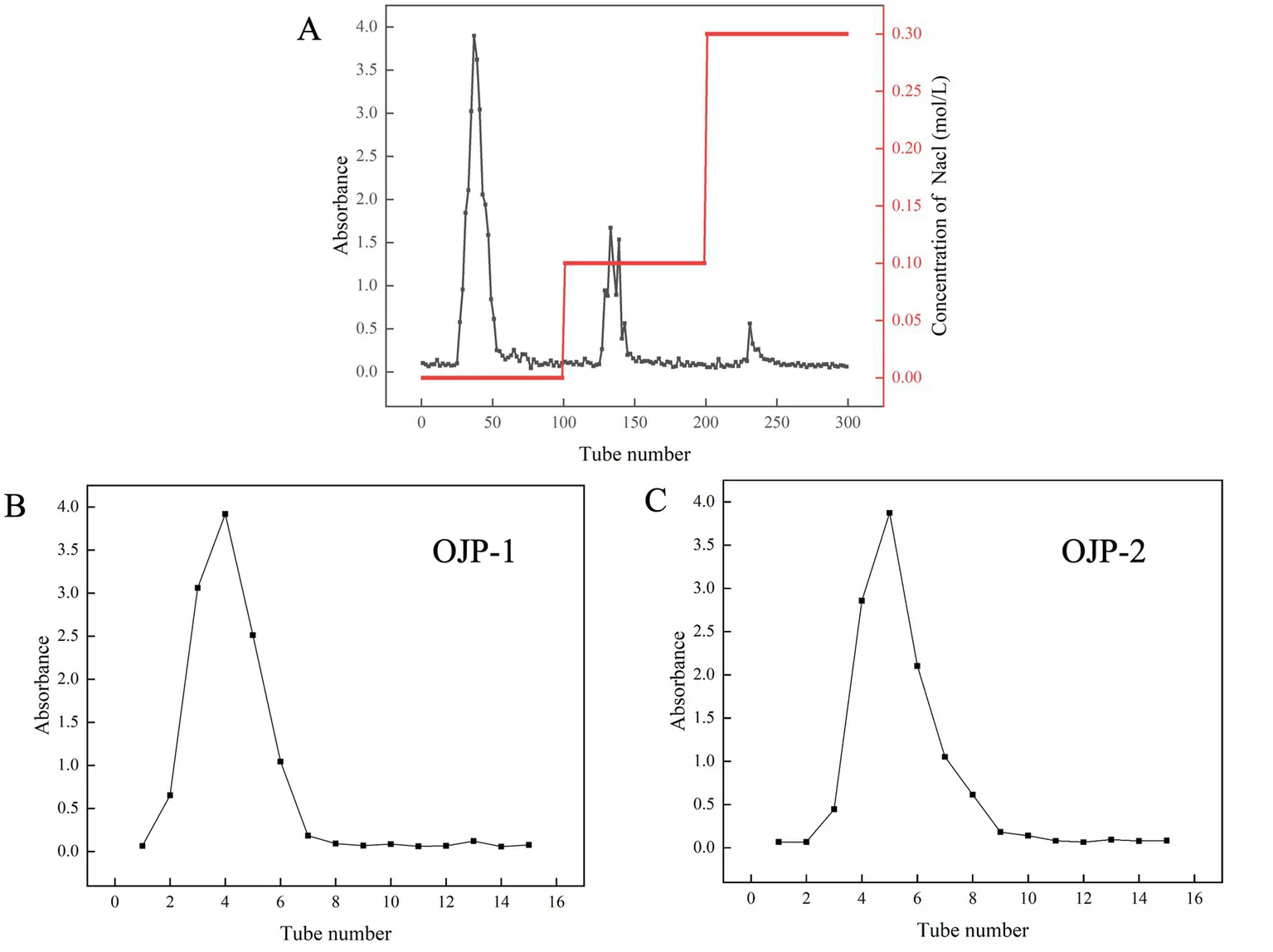
Elution curves of OJP on a DEAE-52 column with different NaCl concentration gradients (NaCl concentrations on the right *y*-axis: 0, 0.1, and 0.3 M) **(A)**; elution curve of OJP-1 on a Sephadex G-100 column **(B)**; elution curve of OJP-2 on a Sephadex G-100 column **(C)**.

Crude OJPs were separated into three components by DEAE-52 cellulose column chromatography (Figure 3A), and the first two components were further purified by Sephadex G-100 chromatography. The preparative Sephadex G-100 elution profiles (Figures 3B,C) each showed one main collected peak under the fractionation conditions. OJP-1 had a white, sparse, layered appearance, whereas OJP-2 had a white, fluffy appearance. The component eluted with distilled water (OJP-1) had the highest yield. Water molecules interact with polysaccharides through hydrogen bonding, and hydration behavior can depend on polysaccharide structure (Fringant et al., 1996; Grossutti and Dutcher, 2016). These hydration principles are relevant to the aqueous solubility of the fractions.

### 3.4 Chemical composition analysis

Both fractions were obtained after purification and had high total sugar contents. Protein and uronic acid were not detected by the Coomassie Brilliant Blue and carbazole-sulfuric acid assays, respectively.

### 3.5 Structural characterisation

#### 3.5.1 UV–vis and FT-IR spectroscopy analyses

**Figure 4:**
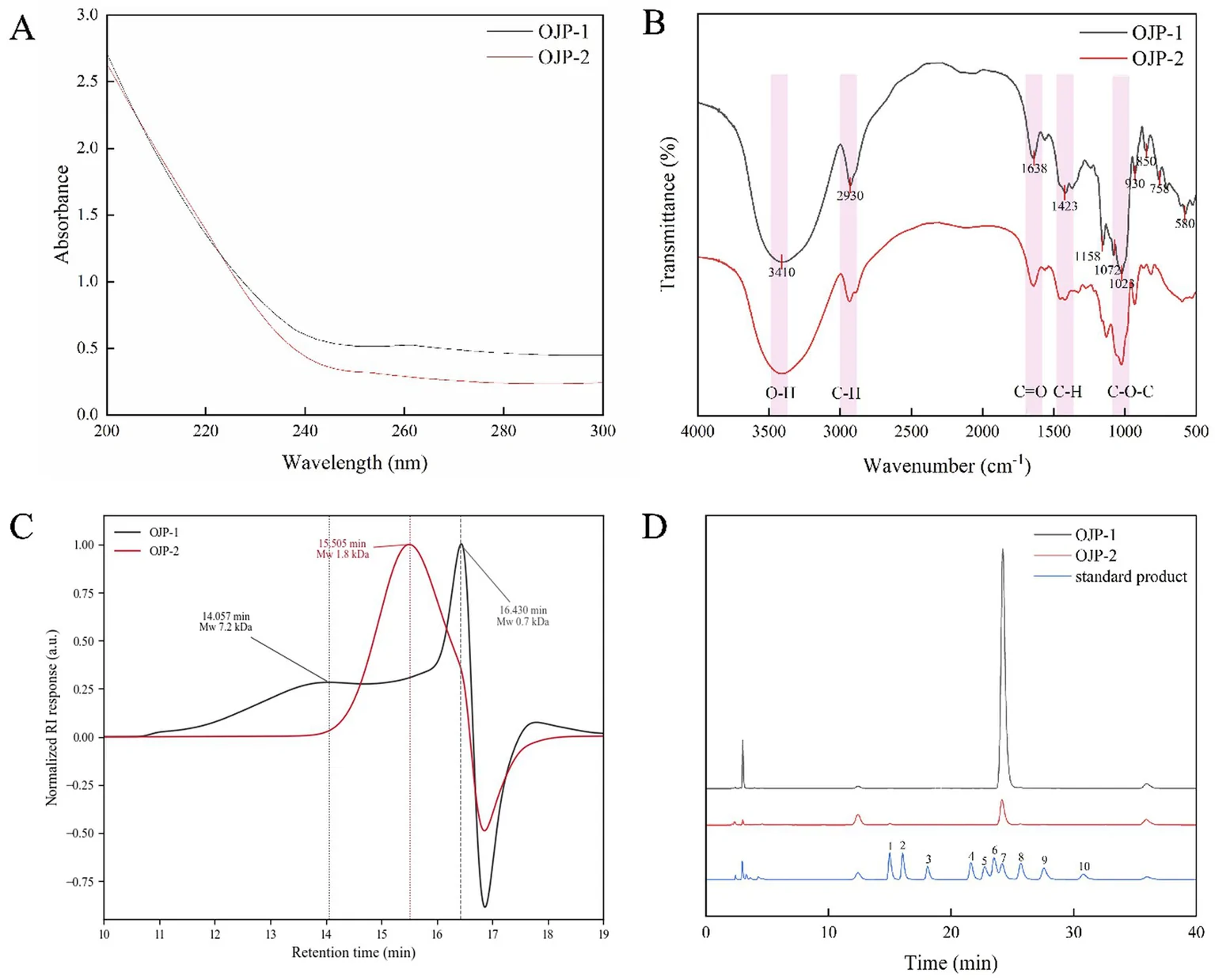
Structural characterisation of OJP-1 and OJP-2. **(A)** UV–vis spectra; **(B)** FT-IR spectra; **(C)** HPGPC chromatograms and peak-resolved molecular-mass profiles; peak labels show retention time (min) and *Mw* (kDa); **(D)** monosaccharide-composition chromatograms (1: Mannose, 2: Glucosamine, 3: Rhamnose, 4: N-acetylglucosamine, 5: Glucuronic acid, 6: Galacturonic acid, 7: Glucose, 8: Galactose, 9: Xylose, and 10: Fucose).

The UV–vis spectra of both fractions showed no distinct absorption peaks at 260 or 280 nm, indicating no detectable nucleic-acid or protein-associated absorbance under the measurement conditions (Figure 4A). This observation was consistent with the Coomassie Brilliant Blue result reported in Section 3.4.

FT-IR can effectively reveal the structural features of polysaccharides. The FT-IR spectra of OJP-1 and OJP-2 are shown in Figure 4B. The stretching vibration of hydroxyl (O-H) was identified as the cause of the strong and broad stretching vibration peak at 3410 cm^−1^. The peak at 2930 cm^−1^ was attributed to the C-H asymmetric stretching vibration. The peak at 1638 cm^−1^ represented the shearing vibration of bound water. The peak near 1,423 cm^−1^ was mainly caused by the C-H bending vibration of the sugar ring and side chain. In addition, the absorption peak at 1023 cm^−1^ was related to C-O-H and C-O-C of the pyranose ring.

#### 3.5.2 HPGPC molecular-mass profiles

The HPGPC results showed a principal OJP-2 peak at 15.505 min (Mw = 1.8 kDa). OJP-1 showed two peaks at 14.057 min (Mw = 7.2 kDa) and 16.430 min (Mw = 0.7 kDa) (Figure 4C). Thus, OJP-1 exhibited a bimodal molecular-mass profile, whereas OJP-2 displayed a single principal peak.

The HPGPC chromatograms showed distinct molecular-mass profiles for the two fractions. Related studies have reported biological activities of structurally characterized polysaccharides (Huo et al., 2021) and structural and immunomodulatory properties of a *Nostoc commune* polysaccharide (Pan et al., 2024). Together with the monosaccharide data, the HPGPC molecular-mass profiles show that OJP-1 and OJP-2 are structurally different fractions. These differences accompanied their distinct activity responses.

#### 3.5.3 Monosaccharide composition analysis

Monosaccharide composition differed between the fractions. OJP-1 was mainly composed of glucose, whereas OJP-2 was composed of glucose, galactose, and xylose in a molar ratio of 92.35:2.33:2.88 (Figure 4D). Differences in monosaccharide proportions have been associated with differences in polysaccharide properties and bioactivities (Chen et al., 2026). No peaks corresponding to uronic acid were observed in the sample chromatograms at the retention positions of the uronic-acid standards; under the chromatographic conditions used, both fractions were therefore characterized as neutral polysaccharides without detectable uronic acid.

A comparative study of jujube polysaccharides obtained by hot-water, ultrasound-assisted, enzyme-assisted, and ultrasound-assisted enzymatic extraction found that the combined ultrasound-assisted enzymatic treatment at 22/33 kHz yielded significantly higher galactose, arabinose, and rhamnose contents than the other extraction methods (Guo et al., 2024). This result illustrates how extraction conditions can shape monosaccharide composition.

#### 3.5.4 SEM analysis

**Figure 5:**
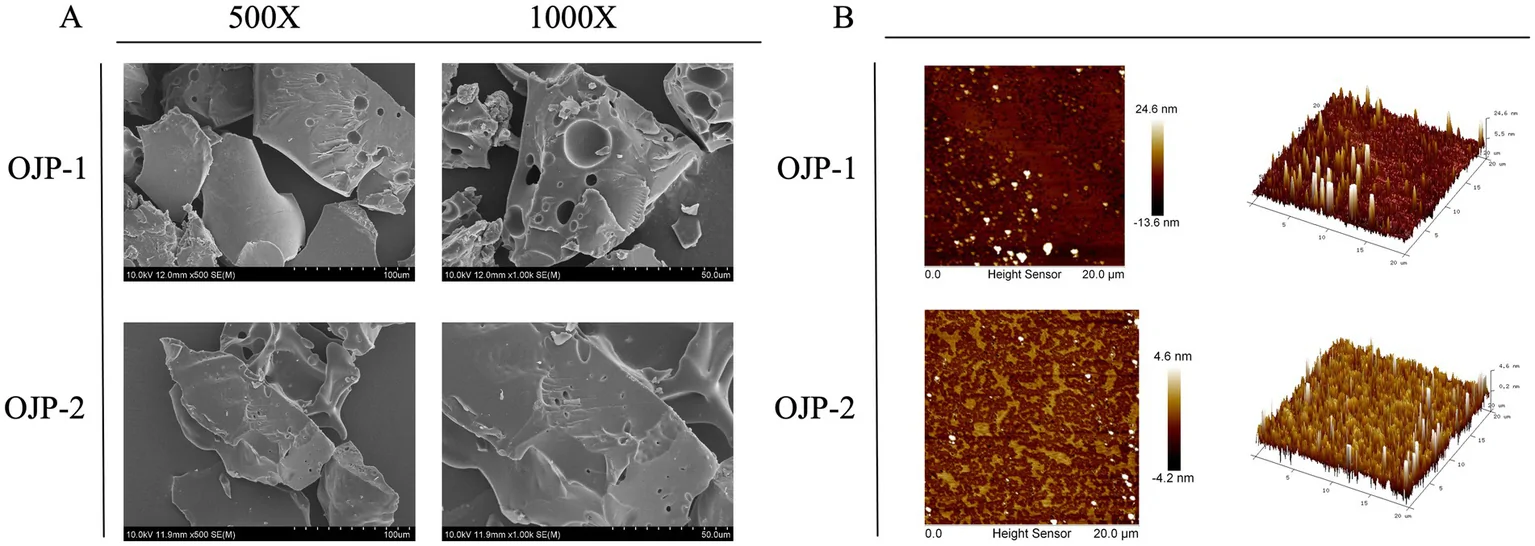
SEM images **(A)** and AFM images **(B)** of OJP-1 and OJP-2.

Both OJP-1 and OJP-2 exhibited irregular block-like or sheet-like aggregates (Figure 5A). OJP-2 was looser and more porous than OJP-1, whereas OJP-1 showed denser local aggregation. Layered surface morphology has also been reported for a structurally characterized dextran from *Imperata cylindrica* (Yu et al., 2023). Enzyme-associated surface changes have been described in other polysaccharide systems (Hu et al., 2025). The SEM images distinguish the morphology of OJP-1 and OJP-2.

#### 3.5.5 AFM analysis

AFM showed irregular nanomound-like deposition for both fractions (Figure 5B). The maximum observed heights were 24.6 nm for OJP-1 and 4.6 nm for OJP-2, and OJP-2 showed a more dispersed, irregular aggregation pattern. Frequency-dependent ultrasound-associated changes in polysaccharide chain conformation have been reported (Hua et al., 2022). The AFM images distinguish the topographic profiles of OJP-1 and OJP-2.

#### 3.5.6 Triple-helix structure analysis

**Figure 6:**
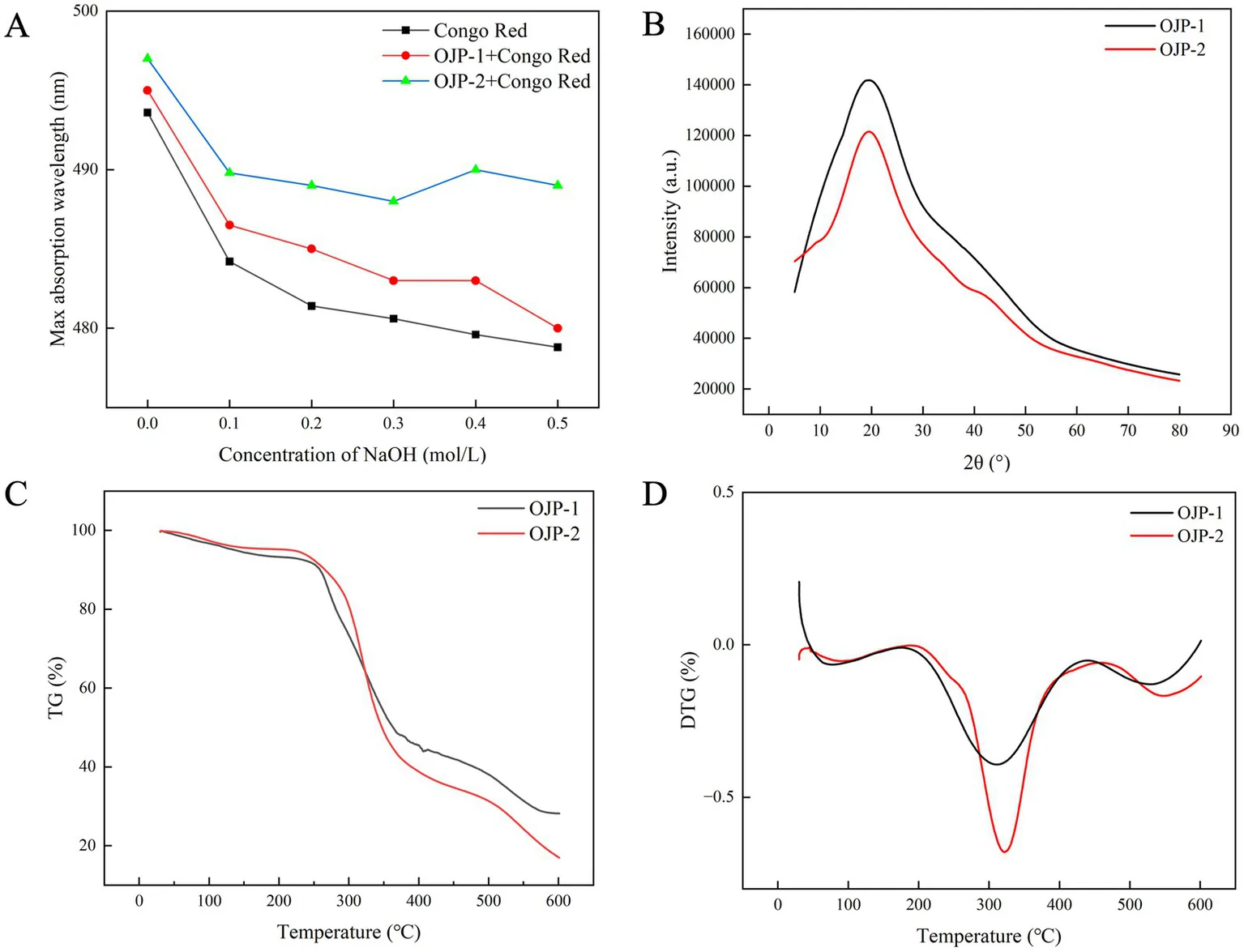
Congo Red test results **(A)**, XRD **(B)**, TG curve **(C)**, and DTG curve **(D)** of OJP-1 and OJP-2.

Sugars with a triple-helix structure can form complexes with Congo red, leading to an increase in their maximum absorption wavelength (Yang et al., 2023). OJP-1 and OJP-2 did not show any significant red shift (Figure 6A). With an increase in sodium hydroxide concentration, the maximum absorption wavelength for both showed a trend similar to that of the blank control, indicating the absence of a triple-helix structure.

#### 3.5.7 XRD analysis

XRD is an effective technique for deciphering the crystal structure of polysaccharides. Both OJP-1 and OJP-2 exhibited broad and diffuse diffraction peaks in the range of 2θ = 15–30°, with no obvious sharp crystal diffraction peaks, indicative of an amorphous structure (Figure 6B).

### 3.6 Thermal stability analysis

As shown in Figures 6C,D, OJP-1 and OJP-2 showed a slight loss in weight at 30–120 °C, mainly due to the evaporation of adsorbed and bound water in the polysaccharide molecules (Tang et al., 2021); a major weight loss occurred at 200–350 °C, corresponding to the cleavage of glycosidic bonds and thermal degradation of the polysaccharide backbone structure (Fang et al., 2020); at temperatures beyond 350 °C, the weight loss rate decreased significantly, and the sample gradually carbonized and formed a stable residue. In contrast, the initial decomposition temperature and maximum weight loss rate temperature for OJP-1 were slightly higher than those for OJP-2, indicating greater thermal stability of OJP-1. Ultrasound-assisted extraction has been associated with altered thermal properties in other polysaccharide systems (Wen et al., 2025).

### 3.7 Antioxidant activity analysis

#### 3.7.1 ABTS scavenging ability

**Figure 7:**
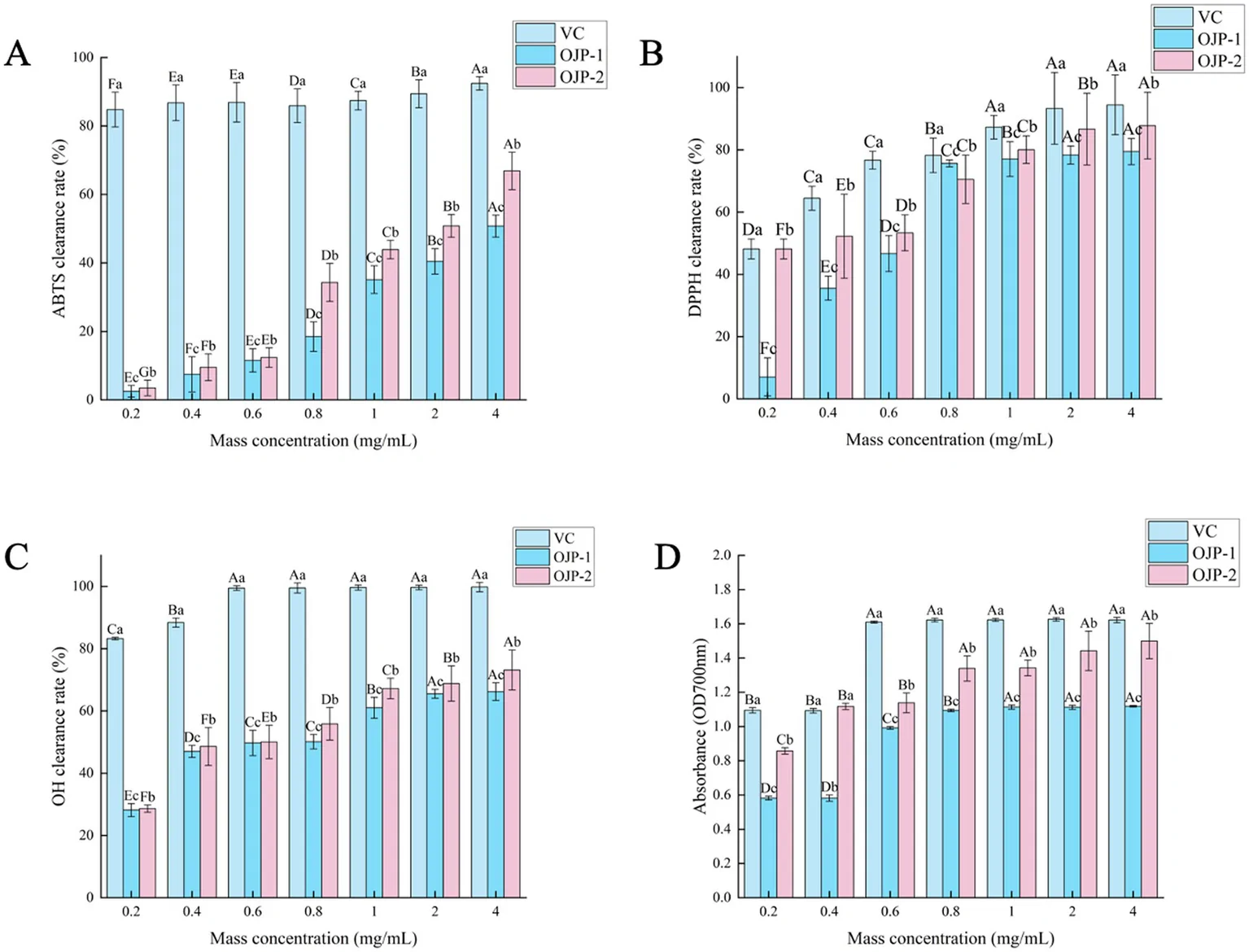
Scavenging activity and reducing capacity (D) of the two polysaccharides against ABTS free radical **(A)**, DPPH free radical **(B)**, and hydroxyl free radical **(C)**. Different uppercase (lowercase) letters indicate significant differences within (between) groups, i.e., *p* < 0.05.

OJP-2 showed stronger ABTS radical-scavenging activity than OJP-1 (Figure 7A). Its distinct HPGPC molecular-mass profile, monosaccharide composition, and morphology accompanied this activity difference. Similar ABTS-scavenging activities have been reported for sunflower-derived pectin (Ezzati et al., 2020) and fig-skin pectin (Gharibzahedi et al., 2019). Antioxidant activity is influenced by multiple structural and compositional features (Zhong et al., 2020).

#### 3.7.2 DPPH scavenging ability

The DPPH radical, a stable free radical with a characteristic absorption band at 517 nm, is commonly used to evaluate antioxidant capacity (Maingam et al., 2024). At 4 mg/mL, OJP-1 and OJP-2 showed DPPH radical-scavenging rates of 79 and 88%, respectively, and their EC50 values were 0.62 and 0.29 mg/mL, respectively (Figure 7B). Thus, OJP-2 displayed greater DPPH radical-scavenging activity. Ultrasound-extracted polysaccharides from other sources have also shown antioxidant activity (Tang et al., 2024), consistent with the stronger DPPH response observed for OJP-2.

#### 3.7.3 •OH-scavenging ability

Hydroxyl-radical scavenging is commonly included in antioxidant evaluation of polysaccharide preparations (Wang et al., 2021). The scavenging responses of both fractions increased with concentration (Figure 7C). At 4 mg/mL, OJP-1 and OJP-2 showed scavenging rates of 66 and 73%, respectively, and their EC50 values were 0.74 and 0.60 mg/mL, respectively. OJP-2 therefore showed greater hydroxyl-radical-scavenging activity than OJP-1. Studies of polysaccharides from guarana, *Acanthopanax senticosus* leaves, and jujube have also reported hydroxyl-radical-scavenging activity (Dalonso and de Oliveira Petkowicz, 2012; Xia et al., 2020; Ji et al., 2020).

#### 3.7.4 Fe^3+^ reducing power

Reducing power reflects electron-donating capacity. The reducing responses of both fractions increased with concentration (Figure 7D). At 4.0 mg/mL, the absorbance values for OJP-1 and OJP-2 were 1.1 and 1.5, respectively, and OJP-2 showed greater reducing power. This result is consistent with the broader antioxidant pattern observed for OJP-2.

Across all four assays, both OJP-1 and OJP-2 showed concentration-dependent *in vitro* antioxidant activity, and OJP-2 consistently produced the stronger response. This activity difference was accompanied by distinct HPGPC molecular-mass profiles, monosaccharide compositions, morphologies, and thermal behaviors.

### 3.8 Simulated *in vitro* digestion (OJP)

#### 3.8.1 Effect of artificial saliva on polysaccharide hydrolysis

Simulated saliva and gastric digestion assays are used to evaluate polysaccharide stability and digestibility under gastrointestinal-like conditions (Yan et al., 2022; Yuan et al., 2020; Yu et al., 2024). After 6 h in artificial saliva at pH 4, 5, 6, 7, and 8, the hydrolysis values for OJP-1 were 3.33, 3.94, 4.77, 4.89, and 4.82%, respectively; the corresponding values for OJP-2 were 3.91, 4.35, 4.54, 5.07, and 4.85%. The pH-dependent differences were small, indicating similar hydrolysis behavior across the tested conditions.

#### 3.8.2 Effect of artificial gastric juice on polysaccharide hydrolysis

After 6 h in simulated gastric fluid at pH 1, 2, 3, 4, and 5, the hydrolysis values for OJP-1 were 2.40, 2.04, 1.67, 1.52, and 1.28%, respectively, whereas those for OJP-2 were 4.41, 4.34, 3.81, 3.63, and 3.28%. The maximum hydrolysis measured for either fraction remained below 5% across the tested gastric pH range.

**Figure 8:**
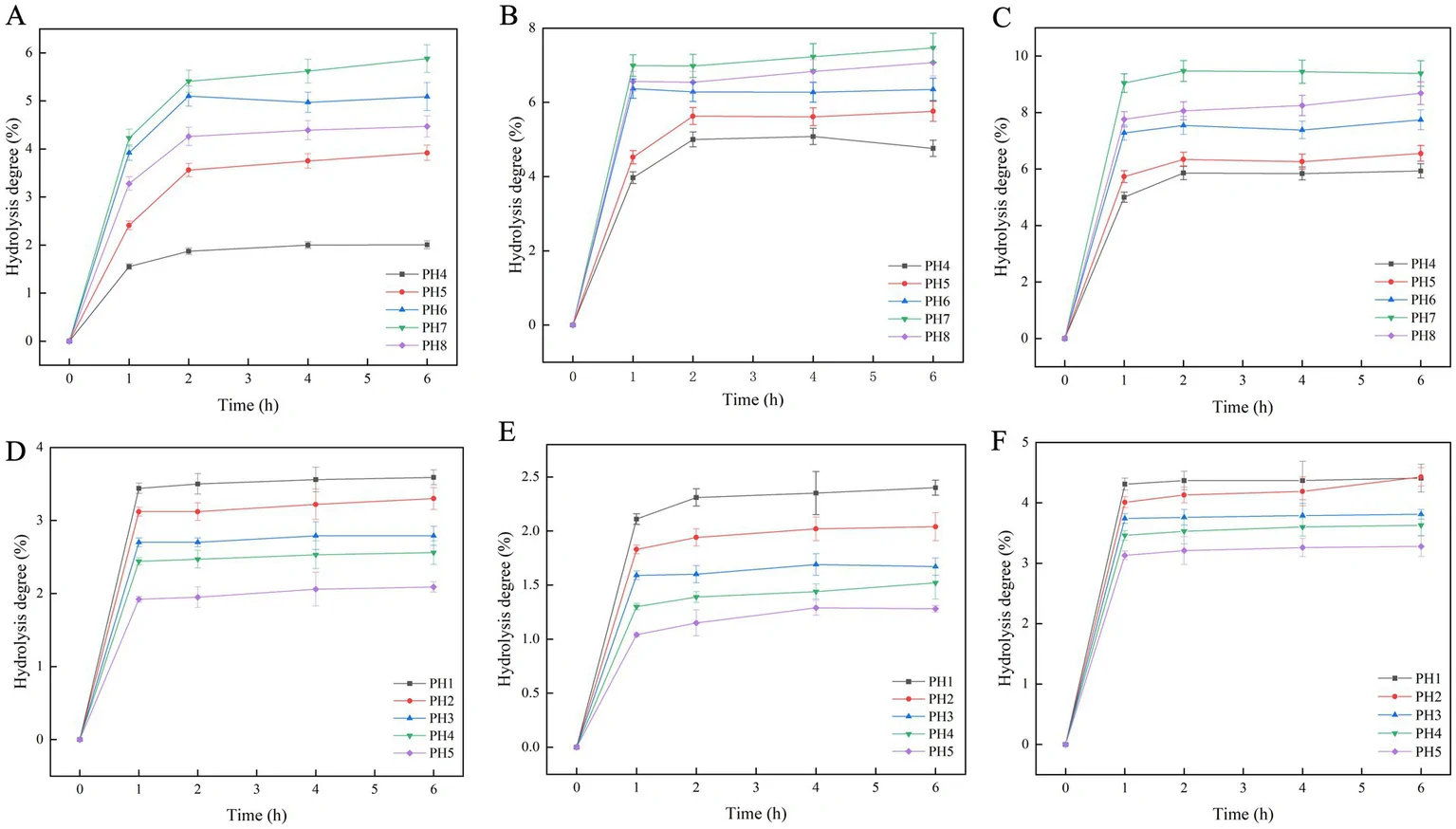
Effects of artificial saliva and artificial gastric juice on polysaccharide hydrolysis. **(A)** Effect of artificial saliva on OJP-1 hydrolysis. **(B)** Effect of artificial saliva on OJP-2 hydrolysis. **(C)** Effect of artificial saliva on FOS hydrolysis. **(D)** Effect of artificial gastric juice on OJP-1 hydrolysis. **(E)** Effect of artificial gastric juice on OJP-2 hydrolysis. **(F)** Effect of artificial gastric juice on FOS hydrolysis.

After 6 h, hydrolysis did not exceed 5.07% in either simulated digestion system. Both fractions therefore resisted hydrolysis under the tested conditions. Similar digestion-related behavior has been reported for polysaccharides in other systems (Luo et al., 2025; Ding et al., 2017; Chen et al., 2016) (Figure 8).

### 3.9 Prebiotic characteristics

#### 3.9.1 Effect of polysaccharide addition amount on probiotic growth curve

**Figure 9:**
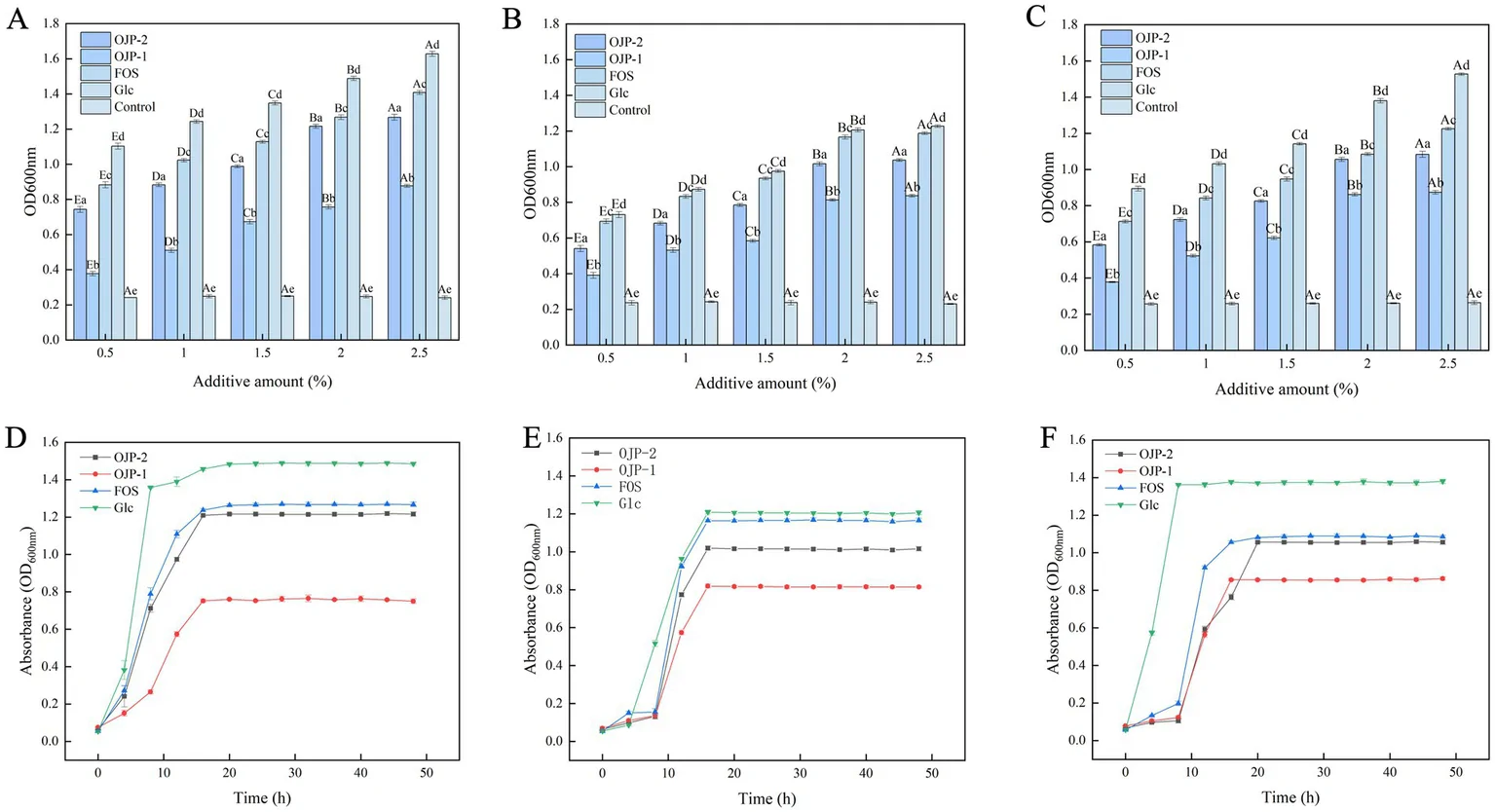
Relationship between probiotic growth curves and optimal polysaccharide addition amount. **(A)** Optimal polysaccharide addition amount for *L. plantarum*. **(B)** Optimal polysaccharide addition amount for *L. acidophilus*. **(C)** Optimal polysaccharide addition amount for mixed cultures. **(D)** Growth curve of *L. plantarum*. **(E)** Growth curve of *L. acidophilus*. **(F)** Growth curve of mixed cultures.

As shown in Figures 9A–C, OJP-1, OJP-2, FOS, and glucose produced significant growth-promoting responses in *L. plantarum*, *L. acidophilus*, and mixed cultures across 0.5–2.0%. Growth promotion increased across this range and reached a plateau at 2.0–2.5%; therefore, 2.0% was selected as the addition level for subsequent experiments.

**Figure 10:**
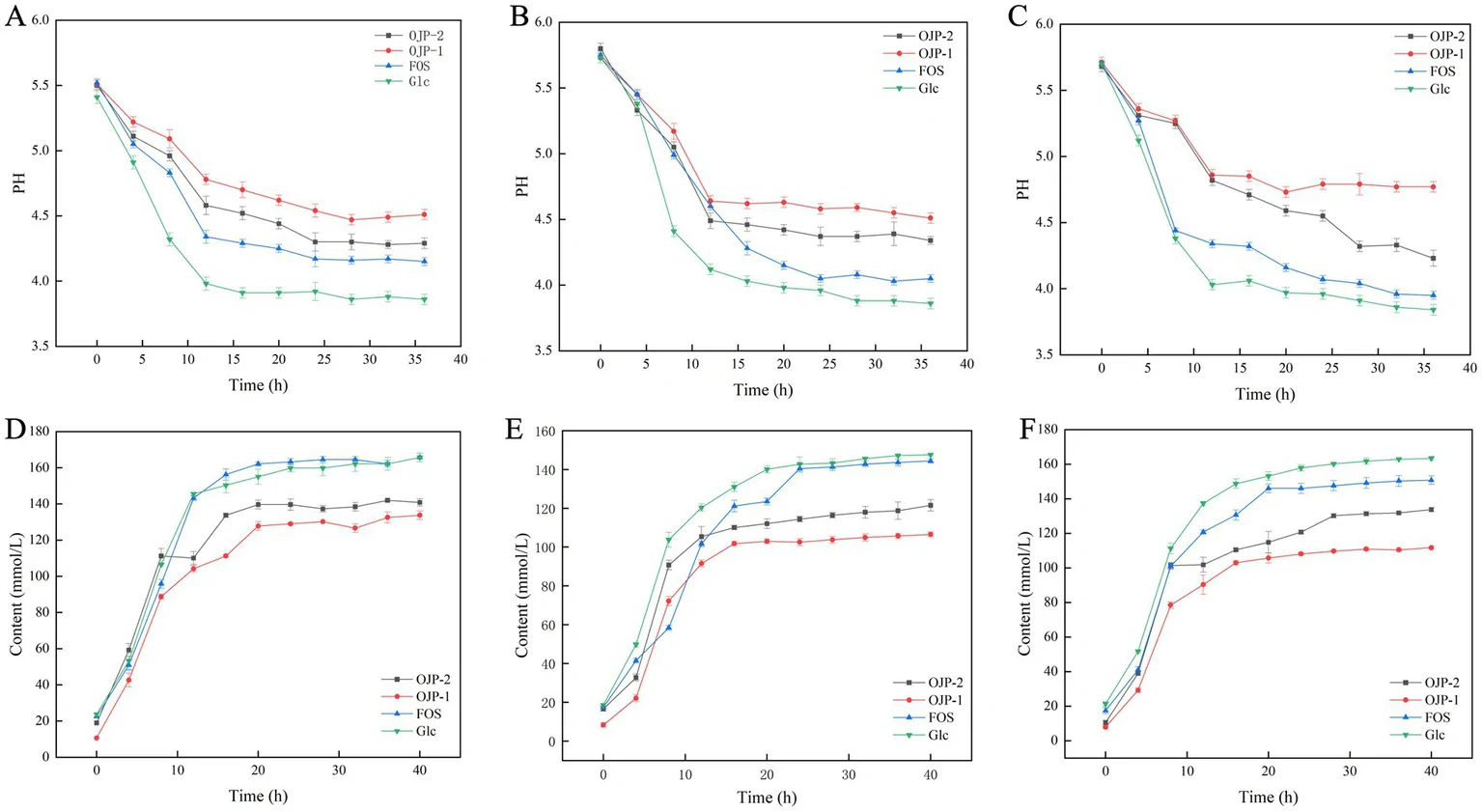
Changes in pH and lactic acid content during probiotic culture. **(A)** Changes in pH during *L. plantarum* culture. **(B)** Changes in pH during *L. acidophilus* culture. **(C)** Changes in pH during mixed culture. **(D)** Changes in lactic acid content during *L. plantarum* culture. **(E)** Changes in lactic acid content during *L. acidophilus* culture. **(F)** Changes in lactic acid content during mixed culture.

Optical density at 600 nm (OD600) was used as an indirect measure of bacterial growth. Compared with the control, the glucose, FOS, OJP-1, and OJP-2 groups showed increased OD600 values from 12 to 24 h, followed by a plateau. The response was strain-dependent, with the order *L. plantarum* > mixed culture > *L. acidophilus*. In the *L. plantarum* and mixed-culture systems, the OD600 values in the OJP-2 and FOS groups were not significantly different (Figures 9D–F). Prebiotic activity has also been reported for a structurally characterized polysaccharide (Fei et al., 2024), while the general prebiotic concept and source-specific activity of bamboo polysaccharides and mulberry-leaf oligosaccharides are described in related studies (Manning and Gibson, 2004; Chen et al., 2020; Hu et al., 2022). OJP-2 produced the stronger growth-promoting response and differed from OJP-1 in HPGPC molecular-mass profile, monosaccharide composition, and morphology.

The structural differences between OJP-1 and OJP-2 were consistent across HPGPC, monosaccharide analysis, SEM, and AFM.

#### 3.9.2 Analysis of pH changes and lactic acid production during probiotic proliferation

Medium pH was used to track culture acidification. pH declined most rapidly during the first 12 h and continued to decline after 12 h in some cultures.

Lactic acid concentrations were measured alongside OD600 in the bacterial culture medium using a lactic acid assay kit. During the early culture stage (0–12 h), lactic acid content in all three culture systems increased over time. After 18 h, the lactic-acid accumulation rate slowed.

Together, the OD600 and lactic-acid results show that both fractions supported probiotic growth *in vitro*.

## 4 Conclusion

This study optimized a sequential two-stage process comprising enzymatic pretreatment followed by simultaneous microwave–ultrasound-assisted extraction of *Ophiopogon japonicus* polysaccharides using response surface methodology. The unrounded model optimum predicted a crude polysaccharide extract yield of 43.999%. Five repeated extractions at the practical rounded settings yielded 43.57 ± 0.26% (mean ± SD; *n* = 5), closely agreeing with the fitted prediction. OJP-1 and OJP-2 differed in monosaccharide composition, microscopic morphology, thermal behavior, and HPGPC molecular-mass profiles. Both fractions exhibited radical-scavenging and probiotic growth-promoting activities, and OJP-2 consistently produced the stronger response. Both fractions resisted hydrolysis in the simulated digestion systems. Together, these results identify OJP-1 and OJP-2 as structurally distinct *O. japonicus* polysaccharide fractions with antioxidant and probiotic growth-promoting activities *in vitro*, with OJP-2 showing the stronger overall response.

## Data Availability

The original contributions presented in the study are included in the article/supplementary material, further inquiries can be directed to the corresponding author.
